# The Impact of Hospice Care on Survival and Healthcare Costs for Patients with Lung Cancer: A National Longitudinal Population-Based Study in Taiwan

**DOI:** 10.1371/journal.pone.0138773

**Published:** 2015-09-25

**Authors:** Jui-Kun Chiang, Yee-Hsin Kao, Ning-Sheng Lai

**Affiliations:** 1 Department of Family Medicine, Buddhist Dalin Tzu Chi Hospital, Chiayi, Taiwan; 2 Department of Family Medicine, Tainan Municipal Hospital, Tainan, Taiwan; 3 Department of Allergy, Immunology and Rheumatology, Buddhist Dalin Tzu Chi Hospital, Chiayi, Taiwan; 4 School of Medicine, Tzu Chi University, Hualien, Taiwan; University of Algarve, PORTUGAL

## Abstract

**Background:**

The healthcare costs of cancer care are highest in the last month of life. The effect of hospice care on end-of-life (EOL) healthcare costs is not clearly understood.

**Purpose:**

The purpose of this study was to evaluate the effect of hospice care on survival and healthcare costs for lung cancer patients in their final month of life.

**Methods:**

We adopted Taiwan’s National Health Insurance Research Claims Database to analyze data for 3399 adult lung cancer patients who died in 1997–2011. A logistic regression analysis was performed to determine the predictors of high healthcare cost, defined as costs falling above the 90th percentile. Patients who received hospice cares were assigned to a hospice (H) group and those who did not were assigned to a non-hospice (non-H) group.

**Results:**

The patients in the H group had a longer mean (median) survival time than those in the non-H group did (1.40 ± 1.61 y (0.86) vs. 1.10 ± 1.47 (0.61), p<0.001). The non-H group had a lower mean healthcare cost than the H group (US $1,821 ± 2,441 vs. US $1,839 ± 1,638, p<0.001). And, there were a total of 340 patients (10%) with the healthcare costs exceeding the 90th percentile (US $4,721) as the cutoff value of high cost. The non-H group had a higher risk of high cost than the H group because many more cases in the non-H group had lower costs. Moreover, the risk of high health care costs were predicted for patients who did not receive hospice care (odds ratio [OR]: 3.68, 95% confidence interval [CI]: 2.44–5.79), received chemotherapy (OR: 1.51, 95% CI: 1.18–1.96) and intubation (OR: 2.63, 95% CI: 1.64–4.16), and those who had more emergency department visits (OR: 1.78, 95% CI: 1.24–2.52), longer hospital admission in days (OR: 1.08, 95% CI: 1.07–1.09), and received radiotherapy (OR: 1.33, 95% CI: 1.00–1.78). Lower risks of high health care costs were observed in patients with low socioeconomic status (OR: 0.58, 95% CI: 0.40–0.83), or previous employment (OR: 0.66, 95% CI: 0.47–0.92). After propensity-score matching, the patients of the non-H group had a higher mean cost and a higher risk of high cost. Similar results were obtained from logistic regression analysis in propensity score-matched patients.

**Conclusions:**

The survival of the hospice group was longer than non-H group, and patients in the non-H group were 3.74 times more likely to have high healthcare costs at EOL. The positive predictors for high health care costs were patients who did not receive hospice care, who received chemotherapy and intubation, who had more emergency department visits and longer hospital admission, and who received radiotherapy. Negative predictors were patients who had a low socioeconomic status or previous employment. The issue of how to reduce the high health care costs for patients with lung cancer in the last month of life is a challenge for policy makers and health care providers.

## Introduction

The topic of the end-of-life (EOL) healthcare costs for cancer patients has been frequently discussed and researched primarily using health administrative data since 2000 [1]. A previous study reported that the mean costs of cancer care were highest in the initial period after diagnosis and the final year of life and lower in the continuing phase, demonstrating a U-shaped curve [2]. Care for cancer patients at the EOL accounts for a large proportion of health care resources. Estimates from the United Kingdom have indicated that approximately 20% of hospital bed days are taken up by patients receiving EOL care [3]. In the United States, estimates have indicated that 25% of healthcare costs are related to patients in their final year of life [4]. Regarding disease, Chastek et al. reported that the costs were highest in the last month of life for cancer patients [5]. A recent review paper reported that palliative care was generally less costly than non-palliative care and that in most cases, and the difference in cost was statistically significant [6]. By contrast, Rabow et al. reported that the mean cost for palliative care patients was higher than that for patients in the control group [7]. One of the reasons for the difference in healthcare costs may be the high diversity in palliative care provision models: in Canada, palliative care is mainly incorporated as a consultation team within institutions and in home-care settings; in England, this care is evolving toward integrating approaches from an earlier institutionalized model, and in the United States, hospice care is mainly home-based [8].

Previous studies have shown that health care costs for terminally ill patients at the end of life can be reduced using various hospice programs, such as hospital-based hospice care [9,10], community-based palliative care [10–12], and hospital palliative care consultation [13], and most cost savings are achieved by reducing hospital stay and the use of resources.

In Taiwan, hospice care service models are implemented by hospital-based hospice care units and a home-based hospice care units (whereby the unit provides both inpatient and home care services). Among patients receiving hospice care, 12.4% receive it at home and 87.6% receive it in a hospital [14]. Patients diagnosed with advanced progressive cancer with a prognosis of approximately 6-months of survival are eligible for palliative and hospice care. The assessment criteria applied in this study were in accordance with Ministry of Health regulations. The application of hospice care was assessed by the hospice care team. If patients with terminal illnesses require palliative or hospice services, they must be transferred to a hospice care ward, and patients or their families sign a do-not-resuscitate form. Similar to other inpatients hospice care facilities worldwide, in Taiwan, more manpower is required in a hospice ward than in a general ward, and palliative and hospice care units comprise a multidisciplinary team, including nurses, physicians, social workers, chaplains, and volunteers. Education curricula and training are provided by three organizations: the Taiwan Academy of Hospice Palliative Medicine, Taiwan Association of Hospice Palliative Nursing, and Taiwan Hospice Organization. Nurses, doctors, and social workers require approximately 13 hours of training for the elementary curriculum and 87 hours for the advanced curriculum before being able to practice as hospice care professionals. The service provided by palliative and hospice teams includes not only inpatient services but also home hospice and bereavement services. Another reason for the difference in healthcare cost at EOL may be the differences in health insurance systems. Patients with catastrophic illness certification receive care for their illnesses or their related conditions and do not pay any out-of-pocket expenses for their care. Taiwan’s National Health Insurance (NHI) reimbursement for hospice care is fixed at US$ 142 per day for inpatient hospice care and US $42–48 per home visit. In Taiwan, even though patients with terminal illnesses require hospice services, they can choose hospital-based or home-based hospice care.

Despite advancements in cancer diagnosis, treatment, and survival, cancer remains a leading cause of death [15]. Lung cancer has been the most common cancer worldwide for several decades, and it is the most common cause of cancer-related death (18.2%) [16]. Furthermore, lung cancer is the leading cause of cancer-related deaths in Taiwan [17]. Meanwhile, the percentage of cancer patients who receive aggressive cancer care at the EOL is increasing [18]. Their increased care has a profound effect on the medical system, health providers, and finances of the health insurance system. The costs of treating cancer are likely to increase in the future with the expected increases in cancer prevalence and aggressive systemic chemotherapy or novel target therapies.

Palliative and hospice care is an approach that improves the quality of life of terminally ill patients and their families through pain relief and the solving of other physical, psychosocial, and spiritual problems, according the World Health Organization definition. Hospice care is a popular model for treating terminally ill patients. Previous studies have reported that patients who receive integrated multidisciplinary palliative care demonstrate improved satisfaction [19–21], improved symptom control [7], and reduced use of acute care services [22] compared with those without hospice care. The quality of EOL care is a vital indicator of excellence in cancer care. Certain quality indicators for EOL cancer care have been proposed and validated in the United States [23, 24] and Canada [25, 26]. However, such quality indicators have been used in only a few studies in Taiwan to evaluate the effect of hospice care on the quality of EOL cancer care.

The purpose of this study was to evaluate the effect of hospice care on survival and health care costs for lung cancer patients in their final month of life.

## Subjects and Methods

We used the claims data from Taiwan’s NHI program to investigate the effect of hospice care on the survival of patients with lung cancer as well as on healthcare costs and determine risk factors for high healthcare costs in the patients’ final month of life.

### Data source

We analyzed claims data obtained from the National Health Insurance Research Database (NHIRD) of Taiwan. The National Health Insurance (NHI) program of Taiwan was implemented in March 1995 and is a single-payer national health insurance system that covered up to 99.9% of Taiwan’s residents in 2012 [27]. We analyzed claims data from 1996 to 2012 for 1 million patients randomly sampled from the 23.22 million NHI enrollees in 2000. In Taiwan, patients with cancer must be examined to receive a catastrophic illness certificate. We used the NHIRD to determine patients with lung cancer and the catastrophic illness database to determine terminally ill lung cancer patients receiving hospice care. Patients under 20 years old were excluded. We followed up patients until December 2012 by using Taiwan’s 2000 Longitudinal Health Insurance Database (LHID2000). The claims data included the medical records (inpatient care, outpatient records, and home care) of patients, including those with and without hospice care.

### Identification

The data of the patients were linked to the LHID2000 to obtain the hospital claims data collected from 1997 to 2011. The International Classification of Diseases, Ninth Revision, Clinical Modification (ICD-9-CM) and A codes were used to define lung cancer (162, A101, 162.0, 162.2, 162.3, 162.4, 162.5, 162.8, 162.9, 165.0, 165.8, 165.9). To increase the validity of the diagnosis of diabetes or hypertension, we defined only patients who had three reported diagnoses of diabetes [28] or 2 instances of hypertension [29], which was determined by the ICD-9-CM or A codes for these disease entities in their medical claims.

### Variables

Patients’ characteristics included age, gender, age at death, mean survival years after cancer diagnosis, anticancer treatment (eg, chemotherapy, radiotherapy, and surgery), geographic location [30], socioeconomic status (SES) [31], level of urbanization, previous employment status [32], whether their last admission was at a teaching hospital, and the department of the last hospitalization (Table 1). Comorbid conditions, such as CCI [33] and common comorbidities (eg, diabetes, hypertension, stroke, hepatitis B infection, hepatitis C infection, chronic kidney disease, and hemodialysis) were identified according to the ICD-9-CM codes.

**Table 1:** Demographic characteristics of lung cancer patients in the H group and non-H group before and after matching.

		Before matching			After matching	
Characteristics	Non-H group	H group	p value	Non-H group	H group	p value
	n (%)	n (%)		n (%)	n (%)	
Number of patients (%)	2833(83.3%)	566(16.7%)		1110(66.7%)	555(33.3%)	
Gender			<0.001			0.668
Male	2012(71.0%)	347(61.3%)		698(62.9%)	343(61.8%)	
Female	821(29.0%)	219(38.7%)		412(37.1%)	212(38.2%)	
Age on death (years)	70.06±12.10	68.89±11.99	0.620	69.69±12.17	69.96±11.95	0.749
Mean survival years, after diagnosis	1.10±0.03	1.40±0.07	<0.001	1.41±0.05	1.38±0.07	0.900
Diabetes	307(10.8%)	82(14.5%)	0.017	144(13.0%)	81(14.6%)	0.362
Hypertension	580(20.5%)	171(30.2%)	<0.001	320(28.8%)	164(29.5%)	0.775
Stroke	272(9.6%)	87(15.4%)	<0.001	149(13.4%)	80(14.4%)	0.598
HBV	53(1.9%)	14(2.5%)	0.323	25(2.3%)	12(2.2%)	1
HCV	47(1.7%)	11(1.9%)	0.596	21(1.9%)	11(2.0%)	1
CKD	81(2.9%)	15(2.7%)	0.890	34(3.1%)	15(2.7%)	0.760
Hemodialysis	52(1.8%)	5(0.9%)	0.149	7(0.6%)	5(0.9%)	0.548
Chemotherapy	1563(55.2%)	352(62.2%)	0.002	710(64.0%)	344(62.0%)	0.450
Radiotherapy	1219(43.0%)	309(54.6%)	<0.001	596(53.7%)	298(53.7%)	1
Operation	254(9.0%)	59(10.4%)	0.266	117(10.5%)	56(10.1%)	0.799
CCI			0.502			0.378
≤2	1199(42.3%)	229(40.5%)		481(43.3%)	225(40.5%)	
= 3	459(16.2%)	87(15.4%)		177(15.9%)	84(15.1%)	
>3	1175(41.5%)	250(44.2%)		452(40.7%)	246(44.3%)	
SES						
LSS	2135(75.4%)	398(70.3%)	0.013	789(71.1%)	390(70.3%)	0.732
MSS	594(21.0%)	140(24.7%)	0.050	269(24.2%)	138(24.9%)	0.809
HSS	104(3.7%)	28(4.9%)	0.153	52(4.7%)	27(4.9%)	0.903
Previous employment	1423(50.2%)	259(45.8%)	0.053	491(44.2%)	256(46.1%)	0.465
Geographic region						
Northern	1053(37.2%)	181(32.0%)	0.019	410(36.9%)	181(32.6%)	0.083
Central	892(31.5%)	134(23.7%)	0.001	241(21.7%)	134(24.1%)	0.263
Southern and eastern	870(30.7%)	246(43.5%)	<0.001	454(40.9%)	236(42.5%)	0.527
Urbanization level						
Urban	1382(48.8%)	302(53.5%)	0.047	639(57.6%)	296(53.3%)	0.105
Suburban	1034(36.5%)	174(30.8%)	0.011	299(26.9%)	171(30.8%)	0.106
Rural	417(14.7%)	89(15.8%)	0.518	172(15.5%)	88(15.9%)	0.886
Teaching hospital, yes	1777(62.7%)	330(58.3%)	0.052	677(61.0%)	324(58.4%)	0.313
Last department of service*						
Hospice ward	0	566(100%)		0	555(100%)	
Chest medicine	1301(45.9%)	0		520(46.8%)	0	
Oncology	443(15.6%)	0		196(17.7%)	0	
Internal medicine	395(13.9%)	0		132(11.9%)	0	
Family medicine	63(2.2%)	0		16(1.4%)	0	
Emergency medicine	161(5.7%)	0		71(6.4%)	0	
Others	470(16.6%)	0		175(15.8%)	0	
Total costs** (US dollars)	15355±16133	21368±20722	<0.001	19198±18678	21018±20538	0.182

Abbreviations: CVA, cerebral vascular accident; CKD, chronic kidney disease; HBV, hepatitis B virus; HCV, hepatitis C virus; SES, socioeconomic status; LES, low SES; MES, moderate SES; HES, high SES.

Survival times: from the date of diagnosis to death.

Matching: propensity-score method.

*: the department providing medical care at the last hospitalization.

**: health care costs from lung cancer diagnosis to death.

### Definition of hospice care and health cost

Hospice care group (H group) and non-hospice group (non-H group): Patients with advanced lung cancer were categorized into the H group if they had ever received hospital-based hospice care including inpatient and/or home hospice care as reported on their medical record. Patients with this disease who had not received hospice care were categorized into the non-H group.

Healthcare cost: We calculated each patient’s healthcare costs by summing the inpatient services and outpatient services listed on their claims records. We converted costs according to the U.S. Dollar and New Taiwan Dollar exchange rate in 2006 (US $1.00 = NT $32.53). We referred to a previous study [34] that reported that Medicare patients with health care costs in the 95th percentile consumed 40% of total Medicare costs. In this study, we defined high health care costs as being greater than the 90th percentile, and a consumption of 39.1% of total health care costs by patients in the last month of life.

Quality indicators of EOL cancer care: The clinical effectiveness of hospice care services for adult patients with terminal illnesses and their family caregivers is defined according to symptom control, quality of life, caregiver distress, and satisfaction with care. Previous studies have reported that quality indicators for hospice care include symptoms related to cancer, such as pain, dyspnea, and depression; treatment-associated toxicities (eg, diarrhea, delirium, skin rash); information and care planning (eg, advanced directive or a surrogate decision maker); communication about chemotherapy; and psychosocial care[35–38]. In this study, information on symptom control, communication about chemotherapy, and psychosocial care was unavailable in the NHIRD data; therefore, we used the following indicators to appraise the quality of EOL cancer care. The quality indicators of EOL cancer care in the last month of life are outlined as follows: received chemotherapy within 2 weeks of death, visited more than one emergency department (ED), hospitalized more than once, admitted to at least one intensive care unit (ICU), or died in hospital [23, 24].

Socioeconomic status (SES): According to the procedures described in previous studies [39, 40], we classified SES into 3 groups: low socioeconomic status (LSS) group, comprising patients earning less than US $615 (NT $20,000) monthly; moderate socioeconomic status (MSS) group, comprising patients earning between US $615 and US $1,230 (NT $20,000–40,000) monthly; and high socioeconomic status (HSS) group, comprising patients earning more than US $1,230 (NT $40,000) monthly.

Charlson comorbidity index (CCI): We calculated the CCI by examining the ICD-9-CM diagnosis and procedure codes recorded in the year before diagnosis according to the Deyo method. We subsequently applied the calculated indices to the inpatient and outpatients claims as reported by Klabundle et al. [41–43].

The protocol for this study was reviewed and approved by the Research Ethics Committee of Buddhist Dalin Tzu Chi Hospital, Taiwan (No. B10301001). Because the NHIRD files contained only deidentified secondary data, the review board waived the requirement for informed consent.

### Statistical analysis

All statistical operations were performed using R 3.0.2 software (R Foundation for Statistical Computing, Vienna, Austria). A 2-sided p value ≤ 0.05 was considered statistically significant. The distributional properties of continuous variables were expressed by mean ± standard deviation (SD), and categorical variables were presented by frequency and percentage. The survival duration was defined as the duration from the day of diagnosis to the date of death (in years). Survival probabilities were analyzed using the Kaplan–Meier method and tested using the log rank test. Normality was examined by conducting a Shapiro–Wilk test. In the univariate analysis, the two-sample t test, Wilcoxon rank-sum test, chi-square test, and Fisher exact test were used to examine the differences in the distributions of continuous variables and categorical variables between the 2 groups (i.e., the H and non-H groups).

We conducted a regression analysis in which the patients’ demographic and clinical characteristics including age, gender, CCI, geographic area of residence, and treatment modality (Tables 1 and 2) were assessed. A multivariate analysis was conducted by fitting multiple logistic regression models with the stepwise variable selection procedure to determine vital predictors of high cost (the health care expenditure was higher than the 90th percentile of the total health care expenditure) during the final month of life. Generalized additive models were fitted to detect the potential nonlinear effects of continuous covariates and determine appropriate cutoff points for discretizing continuous covariates if necessary during the stepwise variable selection.

**Table 2:** Comparison of indicators of the quality of EOL care in lung cancer patients in the H and non-H groups in the last month of life before and after matching.

	Before matching			After matching		
Variables	Non-H group	H group	p value	Non-H group	H group	p value
	n (%)	n (%)		n (%)	n (%)	
Number (%)	2833(83.3%)	566(16.7%)		1110(66.7%)	555(33.3%)	
More than one admission	453(16.0%)	140(24.7%)	<0.001	191(17.2%)	136(24.5%)	0.001
More than 14 days hospital stay	1220(43.1%)	319(56.4%)	<0.001	540(48.6%)	314(56.6%)	0.003
ICU admission	34(1.2%)	3(0.5%)	0.188	7(0.6%)	3(0.5%)	1
More than one ED visit	82 (2.9%)	28 (4.9%)	0.018	36(3.2%)	27(4.9%)	0.104
Intubation	142(5.0%)	6(1.1%)	<0.001	52(4.7%)	6(1.1%)	<0.001
Mechanical ventilation	122(4.3%)	10(1.8%)	0.003	43(3.9%)	10(1.8%)	0.026
New onset hemodialysis^a^	27(1.0%)	1(0.2%)	0.072	2(0.2%)	1(0.2%)	1
Death in a hospital	1290(45.5%)	362(64.0%)	<0.001	581(52.3%)	353(63.6%)	<0.001
Aggressive therapy						
Radiotherapy	391(13.8%)	76(13.4%)	0.841	185(16.7%)	75(13.5%)	0.100
Chemotherapy	1427(50.4%)	344(60.8%)	<0.001	674(60.7%)	336(60.5%)	0.958
Operation	75(2.6%)	13(2.3%)	0.772	38(3.4%)	13(2.3%)	0.291
Cost (US dollars) per capita	1821±2441	1839±1638	<0.001	2024±2496	1838±1649	0.004

Abbreviations: TACE: transcatheter hepatic artery chemoembolization; HAIC: hepatic artery infusion chemotherapy; PEI: percutaneous ethanol injection; RFA: radiofrequency ablation; ICU: intensive care unit; ED: emergency department.

^a^ New onset hemodialysis is patients had no hemodialysis history before last month of life.

We assessed the goodness-of-fit of the final logistic regression model according to the estimated area under the receiver operating characteristic (ROC) curve (also called the c statistic). In practice, the value of the *c* statistic (0 ≤ c ≤ 1) ≥ 0.7 suggests an acceptable level of discrimination power. Statistical tools of regression diagnostics including residual analysis, detection of influential cases, and check of multicollinearity were applied to discover any problems associated with regression model or data. For sensitivity analysis, we also performed logistic regression analysis of propensity score-matched patients.

## Results

**Fig 1:**
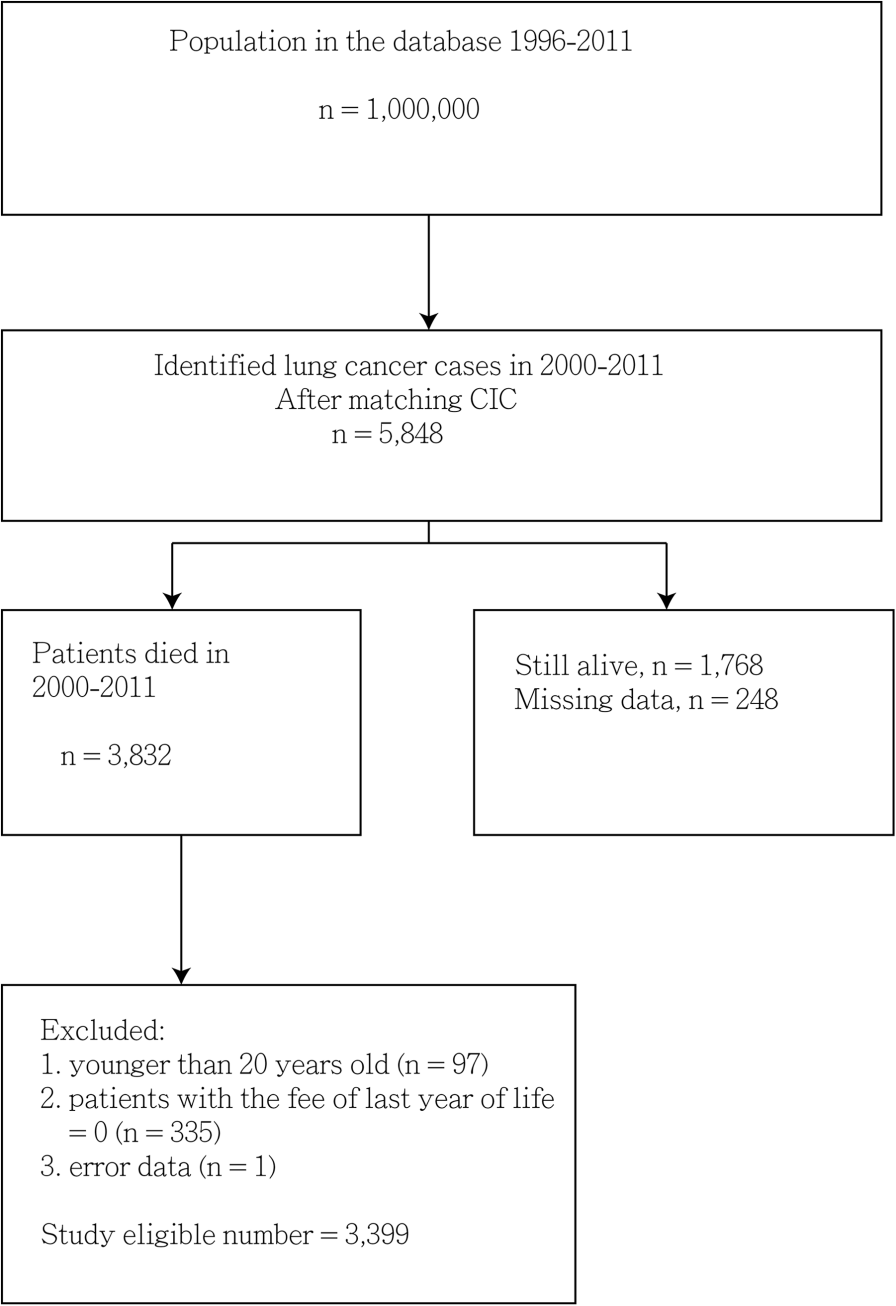
Study flow chart. Abbreviations: ICD-9-CM, International Classification of Diseases, Ninth Revision, Clinical Modification; CIC, catastrophic illness certificate.

**Fig 2:**
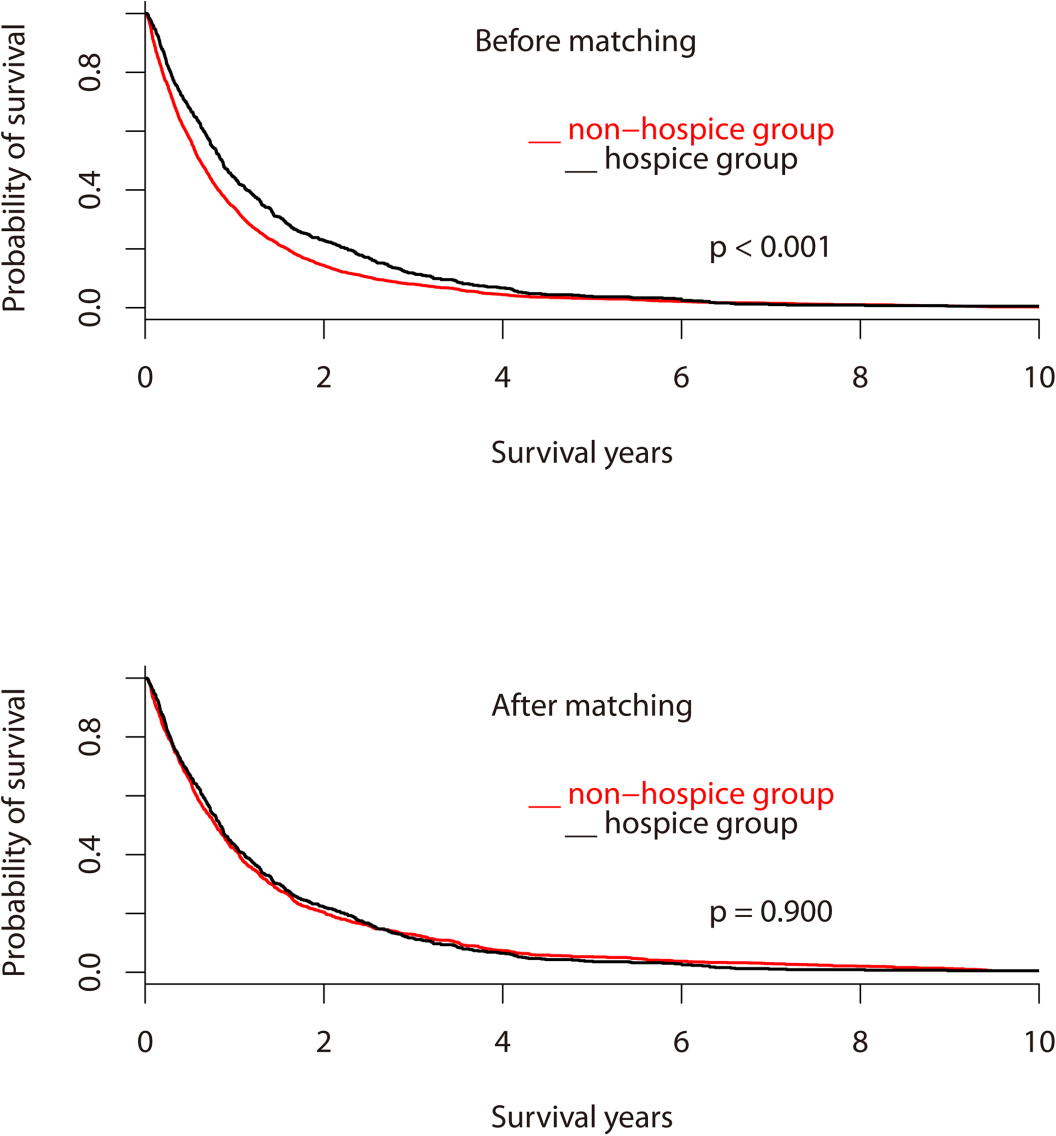
Kaplan-Meier estimates of survival curves for patients with advanced lung cancer stratified into the non-H and H groups.

We enrolled 3399 adult patients (2359 men and 1040 women; ratio = 2.27:1) with lung cancer who died in 1997–2011. Fig 1 depicted the study design. As illustrated in Fig 2, the mean ± SD (median) survival probability in years from diagnosis to death for patients in the H group was higher than that of those in the non-H group (i.e., 1.40 ± 0.07 (0.86) vs. 1.10 ± 0.03 (0.61); log rank test p < 0.001). The mean ± SD (median) days from hospice enrollment to death was 54.7 ± 100.2 (23.0). Furthermore, 239 (42.2%) patients in the H group registered hospice duration of more than 1 month. Table 1 summarizes the characteristics of the sample used in this study. The continuous data (ages, survival years, and cost in the last month of life) did not fit the normal distribution. The patients in the non-H group were more likely to belong to the LSS group (p = 0.013) than those in the H group were. The most common department at the last hospitalization for the non-H group was Chest Medicine, followed by Oncology and Internal Medicine.

**Fig 3:**
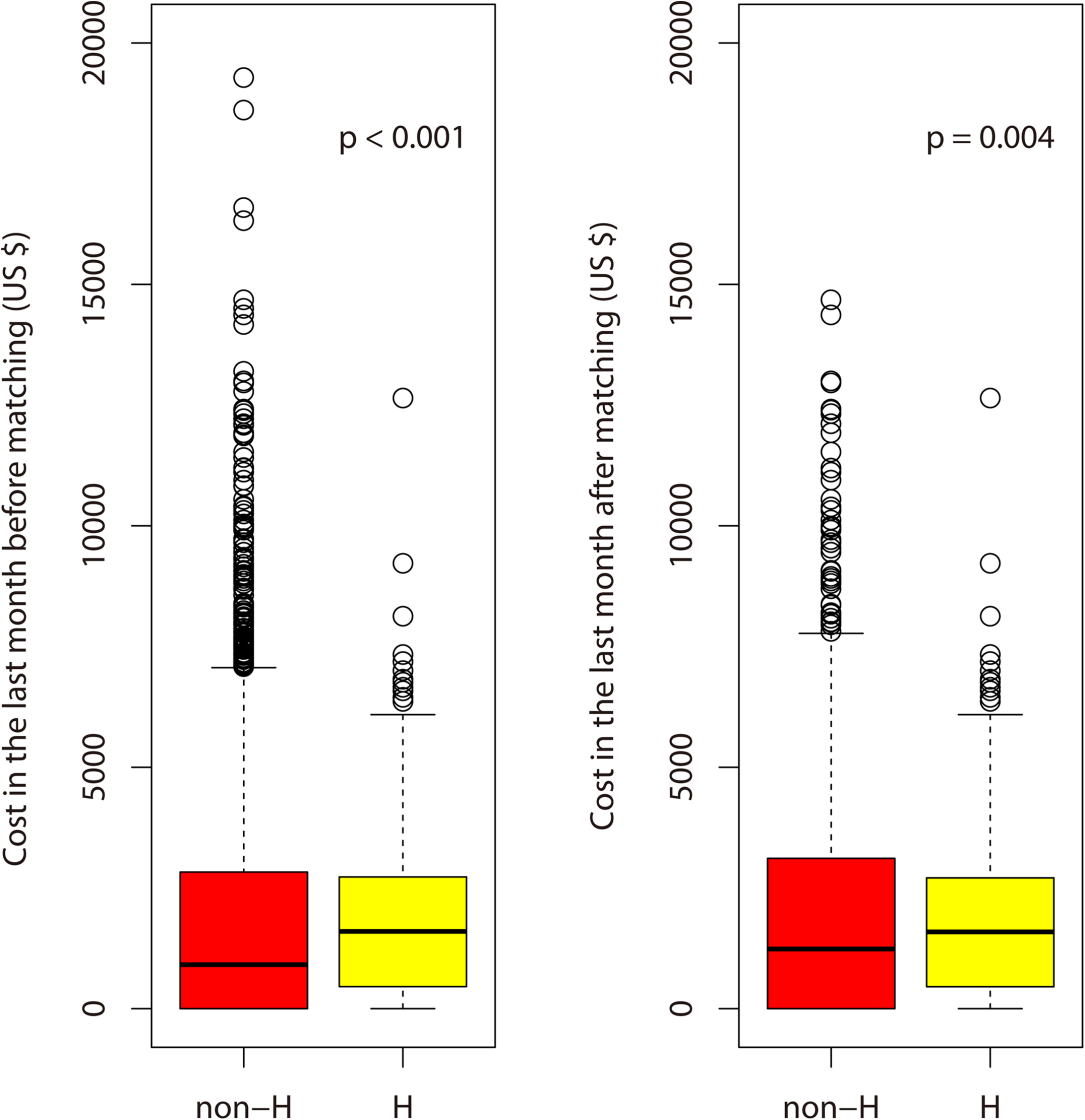
Box plots of costs in the last month of life for patients with advanced lung cancer in the non-H and H groups before and after 2:1 propensity score-matching. The mean values of costs in the last month of life in the non-H and H groups were US $1,821 (median = 910, SD = 2,441) and US $1,839 (median = 1,600, SD = 1,638) before 2:1 propensity-score-matching and US $2,024 (median = 1,236, SD = 2,498) and US $1,838 (median = 1,590, SD = 1,649) after 2:1 propensity-score-matching, respectively.

During the final month of life, comparison of indicators of the quality of EOL care in lung cancer patients in the H and non-H groups was shown in Table 2. The mean values of cost in the last month of life in the non-H and H groups were US $1,821 (median = 910, SD = 2,441) and US $1,839 (median = 1,600, SD = 1,638) before 2:1 propensity score-matching, respectively. The mean cost in the last month of life for the H group was higher than that of the non-H group. The cost did not fit the normality test. The health care costs for 340 patients (10%) were greater than the 90th percentile (US $4,721) in the last month of life, which was the definition of high cost used in this study. They accounted for 39.1% of the total health care costs. The H group comprised fewer patients (25, 4.4%) with higher health care costs than the non-H group (315, 11.1%) (p < 0.001), as shown in Fig 3.

**Fig 4:**
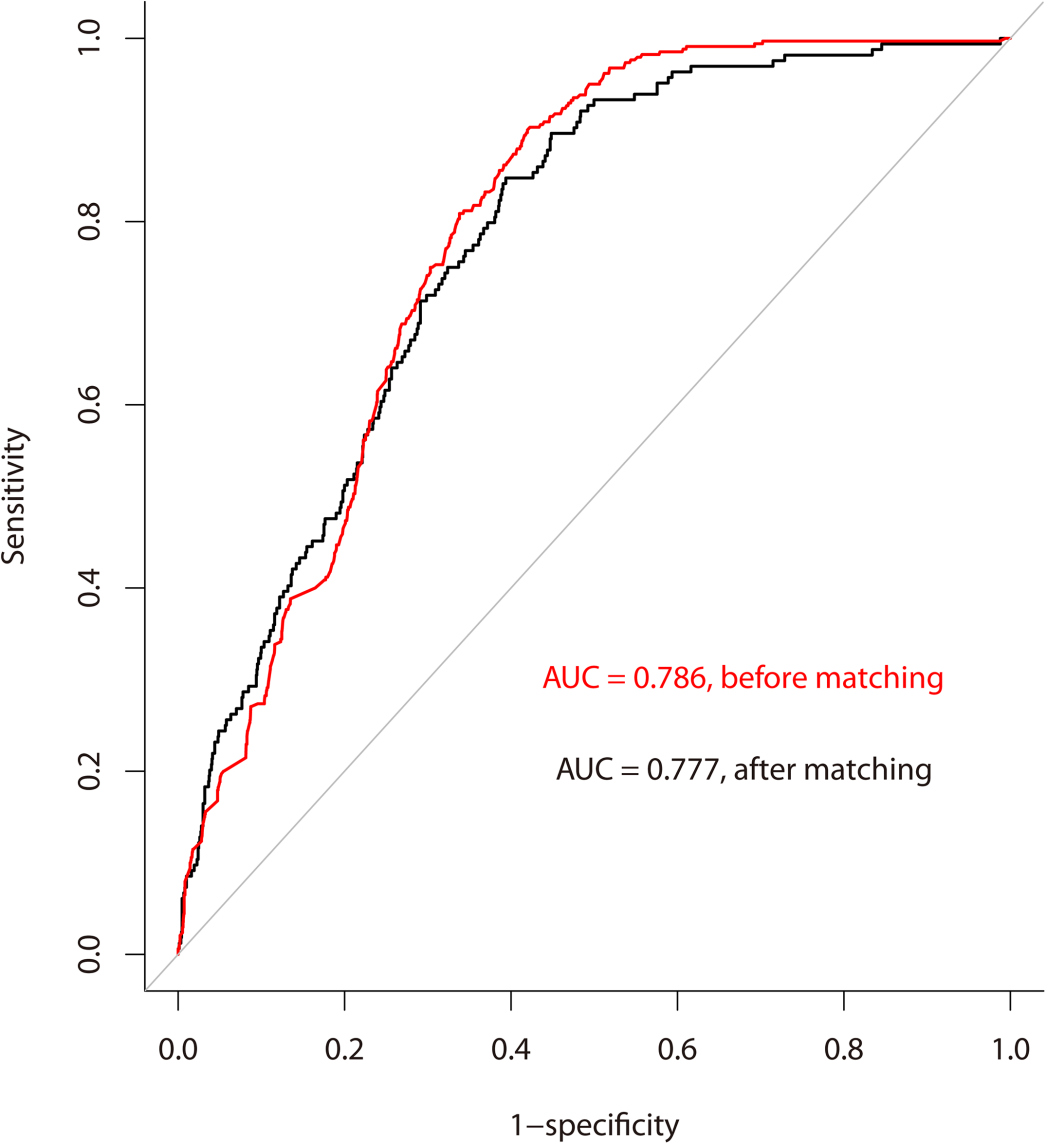
Area under the receiver operating characteristic curve (AUC) was 0.786 for the prediction of high cost (above US $4,721) among patients with advanced lung cancer in their last month of life.

According to our final multiple logistic regression model listed in Table 3, the risk of high healthcare cost in the last month of life was positively associated with non-hospice care (OR = 3.68, 95% CI: 2.44–5.79, p < 0.001), chemotherapy in the last month (OR = 1.51, 95% CI: 1.18–1.96, p < 0.001), use of an endotracheal tube (OR = 2.63, 95% CI: 1.64–4.16, p < 0.001), multiple ED visits (OR = 1.78, 95% CI: 1.24–2.52, p < 0.001), more admission days (OR = 1.08, 95% CI: 1.07–1.19, p < 0.001), and receiving radiotherapy (OR = 1.33, 95% CI: 1.00–1.78, p = 0.050), but negatively associated with patients with low SES (OR = 0.58, 95% CI: 0.40–083, p = 0.003), and previous employment (OR = 0.66, 95% CI: 0.47–0.92, p = 0.017). Although the Nagelkerke R^2^ = 0.189 was not high, the estimated area under the of ROC curve, 0.786 (95% CI: 0.766–0.805), indicated an acceptable level of discrimination power (Fig 4). The R programming code (S1 File) is provided for calculating the probability of high cost based on the final logistic regression model.

**Table 3:** Multivariate Cox proportional hazards model of the factors associated with high health care costs in the last month of life before and after matching.

	Before matching			After matching		
Covariate	O.R.	95% C.I.	p value	O.R.	95% C.I.	p value
Non-hospice (yes vs. no)	3.68	2.44–5.79	<0.001	3.45	2.22–5.55	<0.001
Chemotherapy (yes vs. no)	1.51	1.18–1.96	0.001	1.49	1.01–2.23	0.049
Endotracheal tube (yes vs. no)	2.63	1.64–4.16	<0.001	2.72	1.29–5.45	0.006
Emergency department visit (yes vs. no)	1.78	1.24–2.52	0.001	2.02	1.25–3.20	0.004
Admission days	1.08	1.07–1.09	<0.001	1.07	1.05–1.09	<0.001
Radiotherapy in the last month (yes vs. no)	1.33	1.00–1.78	0.050	1.85	1.24–2.72	0.002
LSS (yes vs. no)	0.58	0.40–0.83	0.003	0.48	0.24–0.90	0.027
Previous employment (yes vs. no)	0.66	0.47–0.92	0.017	0.55	0.29–0.99	0.056
Propensity score				0.80	0.56–1.15	0.236

LSS: low socioeconomic status.

O.R.: odds ratio, C.I.: confidential interval.

In sensitivity analysis, the propensity score was estimated using a multivariate logistic regression model of the non-H group versus the H group conditioning on baseline covariates [44–46]. Patients in the H group were matched with those in non-H group at a ratio of 1:2 by logit (estimated propensity score), using the Matching package in R. After propensity-score-matching, quasi-randomization was observed by examining the balances in covariate distributions between the 2:1 matched non-H and H groups (Table 1). After matching, the mean cost of H group was even lower than non-H group by 9.19% (Table 2). Similar results were obtained from logistic regression analysis of propensity score-matched patients.

## Discussion

The unique finding of this study was that in the last month of life among patients with advanced lung cancer, patients in the non-H group were independently 3.68 times more likely to have high healthcare costs than those in the H group after adjustment.

Previous studies have shown the benefits of various hospice programs. The benefits of early palliative care for advanced lung cancer patients are longer survival, higher quality of life and mood, and less aggressive care at the end of life [47]. The benefits of home hospice care service are that it enables patients to die at home instead of a hospital, avoids hospitalization, and reduces cancer burden [48–51]. A review study reported that patients who received hospice care had greater satisfaction and superior symptom control compared with those who did not receive hospice care [52]. However, hospice care might be associated with longer survival compared with “aggressive” care outside of a hospice. We observed that the hospice care did not associate with shorter survival among patients with advanced lung cancer, and this finding is consistent with those of other studies [47, 53, 54].

In this study, 566 (16.7%) of the patients with lung cancer received hospice care. This rate was similar to that (14.08%) reported by Tang et al., who examined all cancer types in Taiwan [55], but lower than that reported in the United States (35.6%) [5]. Although previous studies have reported that the percentage of cancer patients who received “aggressive” cancer care during the EOL demonstrated an increasing trend, they drew different conclusions regarding the effect of hospice care on the aggressiveness of cancer care [15, 18, 53, 56–59]. Some studies have indicated that hospice care might help attenuate the aggressiveness of cancer care during the EOL [53, 57]. Although Wang et al. reported that Taiwanese cancer patients receiving hospital-based hospice care were significantly less likely to be intubated or use mechanical ventilators; they observed no significant difference in other indicators [56]. Dudgeon et al. reported that the palliative care program reduced ED visits and hospital admissions, even though it did not significantly reduce the percentage of deaths in hospital; but, they did not mention the other 3 QIs included in the current study [58]. Another review study reported that patients who underwent hospice palliative care programs implemented based on hospitals, homes, or outpatient clinics had lower ED visits compared with those who did not participate in such programs [59]. In Taiwan, patients with terminal illnesses requiring hospice service must be transferred to a hospice ward in a hospital for consultation and evaluation. They or their families usually would like to sign a DNR form before receiving hospice care in Taiwan. Oncologists were more likely to recommend palliative chemotherapy or radiotherapy relieving the patients’ pain than primary care providers [60]. These benefits motivate hospice physicians to treat cancer patients closer to death. Another explanation might be insufficient NHI reimbursement to support home hospice care. We suggest that policy makers and health care providers improve accountability in EOL cancer care.

The mean health care costs in the H group were higher than those in the non-H group before matching analysis. This finding differs from previous studies that have reported health care costs as being reduced after hospice care [9–13]. In our stage 1 logistic regression model of H group vs. non-H group for computing propensity score for H group, the predictors included gender, hypertension, stroke, chemotherapy, radiotherapy, hemodialysis, and survival duration. It seemed that the patients of the hospice group were sicker than those of the non-hospice group before propensity score matching. An explanation might be that hospice patients had multiple admissions, hospital stays >14 days, and more ED visits, than those who did not receive hospice care, despite intubation and mechanical ventilation being employed less frequently in the last month of life. Another explanation might be that a lower number of patients in the non-H group had extremely high health care costs, such as those for ICU admission and receiving intubation and mechanical ventilation. However, the mean of health care costs in the non-H group were higher than that in the H-group after propensity-score-matching analysis by 9.19%. The similar finding was reported in previous studies. [61, 62] Although the heath costs were relative saving for patients who chose hospice care, the quality of patients in EOL is our concern.

We observed that patients with advanced lung cancer who had received hospice care had lower mean health care costs in their final month of life compared with those who had not received hospice care after matching. This matching result was consistent with that obtained by Campbell et al., who studied Medicare program payment data and reported that the mean healthcare cost of cancer patients who enrolled into hospice care was, in general, 1% lower than that of patients who did not enroll in hospice care; furthermore, they reported that patients with lung cancer and other aggressive types of cancer who enrolled in hospice care saved 7%–17% more than their counterparts without hospice care did [63]. In the United States, Chastek et al. used medical and pharmacy claims and the Life Sciences Research Database and reported that the mean healthcare cost for cancer patients receiving hospice care in their final month of life was US $2,464 [4]. In the current study, the mean cost for patients receiving hospice care in the last month of life was US $1,838.

A previous study reported that the increase in the healthcare costs in the last month of life was largely due to increased inpatient stay costs [4]. Another study reported that service uptake varied according to gender, marital status, ethnicity, comorbidity burden, insurance status, and geographical location; however, SES or employment was not discussed in this study [1]. In this study, we found that advanced lung cancer patients who belonged to the LSS group or were employed before were less likely to have high costs. We determined that the demographic variable LSS was associated with fewer ED visits, fewer hospitalizations, shorter hospital stays, and fewer deaths in a hospital. A previous study reported that patients with lung cancer after treatment had poorer employment status than the general population [32]. We added the available demographic variables, including previous employment, into our regression analysis as control covariates, and thus we did not intend to make any unwarranted clinical explanation for them. They might be proxies of other unmeasured covariates and this is a limitation of analyzing administrative databases. We also observed that the demographic variable, previous employment was associated with lower rates of ICU care and deaths in hospital. A previous study observed a consistent correlation between higher comorbidity burden and utilization of resources [1].

A previous study reported that thoracic palliative external beam radiotherapy and endobronchial brachytherapy can alleviate thoracic symptoms in patients with metastatic non-small cell lung cancer who are not candidates for curative therapy [64].

Teno et al. revealed that transparency and accountability are required for quality and healthcare costs in EOL care [65]. In Taiwan, hospice patients could receive care services such as ER visits, hospital admission and hospital stay, and anticancer treatment if physicians and patients intend to alleviate symptoms. The healthcare costs of EOL care are transparent in Taiwan because all the reimbursement of healthcare costs at EOL including hospice care was paid from the National Health Institution; furthermore, the reimbursement of hospice care is fixed (US $142 per day for inpatient hospice care and US $42–48 per home visit). We suggest that policy makers promote hospice care programs as well as home hospice care programs as early as possible to improve accountability in health care costs in EOL cancer care.

This study had some limitations. One limitation is the possibility of a misclassification bias because of the accuracy of some of the variables used, including the calculation of the comorbidity score. Another limitation is the fact that patients included in this study were not randomized to the H and non-H groups for comparison. Furthermore, another limitation is that the risk factors related to each quality indicator (eg, clinical symptoms and signs, patient and/or family preferences, and DNR designation) were not recorded in the administrative database. Patient and/or family preference may have influenced some of the outcomes. Although a previous study showed that decisions regarding the cardiopulmonary resuscitation of patients with advanced cancer at EOL were affected by family tradition in China [66], future research is warranted to investigate cultural aspects associated with these preferences in Taiwan. Another limitation was that information on palliative sedation and withdrawal or withholding of certain therapeutics was unavailable in the administrative data.

## Conclusions

Hospice care was associated with longer survival, and patients in the non-H group were 3.68 times more likely to have high health care costs at EOL. After matching, the cost differential would have been 9.19% less in the hospice group. The positive predictors for high health care costs were patients who did not receive hospice care, who received chemotherapy and intubation, who had more emergency department visits and longer hospital stays, and who received radiotherapy; but negative predictors were patients with a low socioeconomic status or previous employment. The issue of how to reduce the high costs for patients with lung cancer in the last month of life is a challenge for policy makers and health providers.

## Supporting Information

S1 File Code for calculating the probability of high cost. (DOC)

## Abbreviations

AUC: area under the receiver operating characteristic curve
CCI: Charlson comorbidity index
CIC: catastrophic illness certificate
CKD: chronic kidney disease
DNR: Do not resuscitate
EOL: end-of-life
ED: emergency department
ICU: intensive care unit
NHI: National Health Insurance
NHIRD: National Health Insurance Research Database
QI: quality indicator
ROC: receiver operating characteristic
SES: socioeconomic status
