# Supplementary material for: The Impact of Hospice Care on Survival and Healthcare Costs for Patients with Lung Cancer: A National Longitudinal Population-Based Study in Taiwan
Source: PLoS One. 2015 Sep 25;10(9):e0138773. doi: 10.1371/journal.pone.0138773 (PMC4583292; doi:10.1371/journal.pone.0138773)
Supplement: S1 File — (DOC) [file pone.0138773.s001.doc]

**Appendix**

**S1 Text. Code for calculating the probability of high cost**

Programming code in OpenOfficeCalc, Microsoft Excel, and R environment for calculating the probability of high cost in the last month based on our multiple logistic regression model.

1. In OpenOfficeCalc or Microsoft Excel:

Key in the values for non-hospice care (non-hospice group= 1, hospice group = 0) in the A1 cell, chemotherapy in the last month (yes = 1, no = 0) in the A2 cell, using endotracheal tube (yes =1, no = 0) in the A3 cell, emergency department visit (yes = 1, no = 0) in the A4 cell, admission days in the last month (days) in the A5 cell, low socioeconomic status (yes =1, no = 0) in the A6 cell, radiotherapy in the last month of life (yes = 1, no = 0) in the A7 cell, and previous employment (yes = 1, no = 0) in the A8 cell. Key in the following formula in any empty cell on the same spreadsheet to obtain the estimated probability of high healthcare costs in the last month of life:

= 1/(EXP(-(-4.53 + 1.30*A1 + 0.41*A2 + 0.97*A3 + 0.58*A4 + 0.08*A5 + (-0.55)*A6 + (0.29)*A7 )+ (-0.42)*A8)+1)

1. In an R environment:

To calculate the probability of high healthcare costs, substitute the values for the variables X1 to X8 in the following regression equation and execute in the R console:

Yhat <- (-4.53 # constant

+ 1.30*X1 # X1= non-hospice (yes=1, no=0)

+ 0.41*X2 # X2 = chemotherapy, (yes=1, no=0)

+ 0.97*X3 # X3 = using endotracheal tube, (yes=1, no=0)

+ 0.58*X4 # X4 = emergency department visiting (yes=1, no=0)

+ 0.08*X5 # X5 = admission days (days)

+ (-0.55)*X6 # X6 = low socioeconomic status,(yes=1, no=0)

+ (0.29)*X7 # X7 = radiotherapy in the last month, (yes=1, no=0)

+ (-0.42)*X8 # X8 = previous employment, (yes=1, no=0)

)

phat <- 1/(1 + exp(-(yhat)))

phat
